# Investigating health system performance: An application of data envelopment analysis to Zambian hospitals

**DOI:** 10.1186/1472-6963-7-58

**Published:** 2007-04-25

**Authors:** Felix Masiye

**Affiliations:** 1 Harvard University Initiative for Global Health, 104 Mount Auburn Street, Cambridge, MA 02138, USA

## Abstract

**Background:**

Zambia has recently articulated an ambitious national health program designed to meeting health-related MDGs. Public expectations are high and Zambia continues to receive significant resources from global and bilateral donors to support its health agenda. Although the lack of adequate resources presents the most important constraint, the efficiency with which available resources are being utilised is another challenge that cannot be overlooked. Inefficiency in producing health care undermines the service coverage potential of the health system. This paper estimates the technical efficiency of a sample of hospitals in Zambia.

**Methods:**

Efficiency is measured using a DEA model. Vectors of hospital inputs and outputs, representing hospital expended resources and output profiles respectively, were specified and measured. The data were gathered from a sample of 30 hospitals throughout Zambia. The model estimates an efficiency score for each hospital. A decomposition of technical efficiency into scale and congestion is also provided.

**Results:**

Results show that overall Zambian hospitals are operating at 67% level of efficiency, implying that significant resources are being wasted. Only 40% of hospitals were efficient in relative terms. The study further reveals that the size of hospitals is a major source of inefficiency. Input congestion is also found to be a source of hospital inefficiency.

**Conclusion:**

This study has demonstrated that inefficiency of resource use in hospitals is significant. Policy attention is drawn to unsuitable hospital scale of operation and low productivity of some inputs as factors that reinforce each other to make Zambian hospitals technically inefficient at producing and delivering services. It is argued that such evidence of substantial inefficiency would undermine Zambia's prospects of achieving its health goals.

## Background

Zambia's health system is poised for a major challenge of executing an ambitious health programme designed towards improving health service delivery and meeting its health-related Millennium Development Goals (MDGs). In 2006, the Ministry of Health (MOH) announced an ambitious national plan to scale-up a range of interventions for fighting the Zambia's leading health problems [1]. Public expectations are high and Zambia continues to receive significant resources from various donors and development agencies to support its health programme. By 2006, the Global Fund for AIDS, Tuberculosis and Malaria (GFATM) alone had given about US$120 million [2]. More resources are likely to be available through the Highly-Indebted Poor Country (HIPC) initiative. These resources will continue to enhance Zambia's capacity to extend coverage of a range of key interventions to its citizens. With total health expenditure currently estimated to be only around US$ 25–30 per capita, there is no doubt that Zambia will need external support in order to further its health agenda [3].

In recent debates about the growing financing needs for health care in Zambia, there has been a preoccupation with mobilising additional resources, particularly from outside. While this focus is truly important, the ultimate solution has to include a broader conceptual perspective. For instance, performance weaknesses that run pervasive in many health systems in Africa render additional money necessary but perhaps not sufficient. A recent study illustrates how organisational and operational weaknesses in a delivery system can thwart service coverage even in situations where financial resources are not the biggest problem [4]. The World Health Report 2000 framework underscored technical efficiency as important in achieving the traditional goals of health systems namely service coverage and possibly population health [5].

In Zambia, hospitals are at the centre of implementing interventions and policies which are crucial to the attainment of the recently articulated health targets. In particular, hospitals provide the largest share of services in antiretroviral therapy (ART), prevention of mother to child transmission of Human Immuno-deficiency virus (PMTC), Tuberculosis treatment, safe deliveries and many other services. Besides their political clout, hospitals are consumers of a substantial proportion of health sector resources. When hospitals consume excess resources in producing their services this invariably results in misallocation and loss of potential care to other beneficiaries. This in turn raises important sustainability and equity implications. Thus, improving efficiency would increase the service potential of existing health infrastructure and provide opportunities for re-allocating resources to other areas.

This paper analyses the technical efficiency of the provision of public hospital services as a key determinant of health system performance. The *sine qua non *of efficiency analysis is measurement, as the very absence of sound evidence about the magnitude and nature of inefficiency precludes any policy action. Efficiency is estimated using a mathematical programming model known as data envelopment analysis (DEA). The paper seeks to achieve the following specific objectives; (i) estimate the productive efficiency of hospitals, (ii) examine the sources of inefficiency, and (iii) explore policy options for improving performance.

## Zambia's hospital institutional context

This section briefly discusses the health financing model and incentives facing hospital decision-makers in Zambia. Both the Government and Mission hospitals are operated by a team of professional management boards under a contracting arrangement with MOH. The idea of hospital boards was to make hospitals more efficient, effective and responsive to their communities. Thus, efficiency in service production and delivery is at least implied. The hospital board members are appointed by the Minister of Health. Each hospital board signs what is referred to as a service contract with MOH which stipulates the type of services to be delivered. The contract also specifies the financial obligations of the Ministry. Although hospitals are supposed to be autonomous in the decision-making, they are still quite heavily regulated [6,7].

In terms of finance, a dual system operates in Zambia. Capital investments and staffing are still the responsibility of MOH while operating costs are covered under a prospective line-item reimbursement system and user-fee revenue. Basically, hospitals are funded on the basis of an annual budget which is based on the cost of inputs. In this context, health financing theory postulates that the incentives for hospitals to operate efficiently are generally weak [8].

Further, the number of beds was determined at the initial planning period implying that this is a relatively non-discretionary quantity as far as hospital decision-makers' influence is concerned. However, hospitals do exercise some discretion to alter the number of beds in any department. Most urban hospitals have now turned some beds into private beds within their hospitals. The reimbursement system is strongly tied to hospital bed capacity, implying that hospitals do have some degree of discretion, albeit limited, over bed-days and related inputs. This is constrained by their limited influence on some inputs and input prices.

Overall, hospital service production is akin to a black-box syndrome. Planners hardly have adequate information about the quantity, quality and even appropriateness of the services for which hospitals are reimbursed. Neither are the true *minimum *costs of producing those services known to the funder (i.e. MOH). This scenario carries a prospect that hospitals would be characterised by operational slack that could undermine health system performance.

Whatever the difficulties of conceptualising hospitals as firms in the traditional economic sense, it is incontrovertible that the decisions that take place in a typical firm of applying a mix of inputs to produce valued outputs also go on in hospitals. It is also beyond doubt that society is concerned about comparing how individual hospitals are performing relative to each other in delivery of care. Finally, the quest for efficient or least-waste health service production is not in conflict with individual clinicians' professional freedom to perform their functions, nor is it an oversight on their clinical standards.

## Methods

Technical inefficiency signals a failure to produce an output with minimum inputs or inability to reach a given output level from available inputs. In either case, inefficiency implies a possibility to obtain greater output and/or save resources. At the service production level, several decisions pertain to operational efficiency of hospitals. These may include selecting the most least-cost inputs (e.g. using nurses instead of doctors or generics instead of brand drugs), minimising excess bed capacity, maximising the effort of staff, eradicating theft of resources, combining available resource inputs in a cost-minimising way, given their prices, and so on. DEA constructs an efficiency frontier which reveals the least input requirement for obtaining a given output level, or, from an output-oriented framework, the highest output obtainable from a given input set.

### The Model

Building on the original work by Farrell [9] on production efficiency, Färe et al [10], showed that technical efficiency can be defined in terms of distance functions. These functions then measure how far an individual observation is from a technology frontier or the 'best practice' production possibility frontier. A novel feature of DEA is that this production possibility or technology set is constructed from observed input-output quantities without specifying a parametric production relation between inputs and outputs. Characterisation of this model involves description of the properties of the technology. First, DEA permits a sample of *N *hospitals (*j *= 1, 2, ..., *N*) each producing *m *outputs (*y*^*j *^= (*y*_1*j*_, *y*_2*j*_, ..., *y*_*mj*_)) from *n *inputs (*x*^*j *^= (*x*_*ij*_, *x*_2*j*_, ..., *x*_*nj*_)). Second, the technology set is convex and allows a (weighted) average input-output bundle. Thus, in a multi-hospital and multi-input-output dimension, the production technology set is described as

L={(x,y):x≥∑j=1Nμjxj;y≤∑j=1Nμjyj;μj≥0; (j=1,2,...,N)}
 MathType@MTEF@5@5@+=feaafiart1ev1aaatCvAUfKttLearuWrP9MDH5MBPbIqV92AaeXatLxBI9gBamXvP5wqSXMqHnxAJn0BKvguHDwzZbqegyvzYrwyUfgarqqtubsr4rNCHbGeaGqiA8vkIkVAFgIELiFeLkFeLk=iY=Hhbbf9v8qqaqFr0xc9pk0xbba9q8WqFfeaY=biLkVcLq=JHqVepeea0=as0db9vqpepesP0xe9Fve9Fve9GapdbaqaaeGacaGaaiaabeqaamqadiabaaGcbaGaeeitaWKaeyypa0Jaei4EaSNaeiikaGIaeeiEaGNaeiilaWIaeeyEaKNaeiykaKIaeiOoaOJaeeiEaGNaeyyzIm7aaabCaeaaiiGacqWF8oqBdaWgaaWcbaGaeeOAaOgabeaakiabbIha4naaCaaaleqabaGaeeOAaOgaaOGaei4oaSJaeeyEaKNaeyizIm6aaabCaeaacqWF8oqBdaWgaaWcbaGaeeOAaOgabeaakiabbMha5naaCaaaleqabaGaeeOAaOgaaOGaei4oaSJae8hVd02aaSbaaSqaaiabbQgaQbqabaGccqGHLjYScqaIWaamcqGG7aWocqqGGaaicqGGOaakcqqGQbGAcqGH9aqpcqaIXaqmcqGGSaalcqaIYaGmcqGGSaalcqGGUaGlcqGGUaGlcqGGUaGlcqGGSaalcqqGobGtcqGGPaqkcqGG9bqFaSqaaiabbQgaQjabg2da9iabigdaXaqaaiabb6eaobqdcqGHris5aaWcbaGaeeOAaOMaeyypa0JaeGymaedabaGaeeOta4eaniabggHiLdaaaa@7F24@

Third, in DEA formulation disposability properties are specified either as free or strong of weak. For freely-disposable inputs, we have the condition that if (*x*^0^, *y*^0^) is feasible, then for any *x *≥ *x*^0^, (x, y^0^) is also feasible. Free disposability implies that inputs do not exhibit negative marginal productivity. In this case, increasing one input while holding other inputs constant will not reduce output. For weakly-disposable inputs, increasing only one input would reduce output (due to negative marginal productivity) unless other inputs are also increased counteractively. Free disposability of outputs means that outputs can be disposed of at no cost. There are cases where production of certain outputs is accompanied by production of 'bads' such as pollution (reducing one output invariably means reducing the other). In such a case, outputs are said to be weakly disposable [10]. In the context of hospital production it is reasonable to assume that both inputs and outputs will be freely disposable.

Finally, the structure of technology is defined by returns-to-scale properties. The shape of the efficiency frontier (and therefore DEA scores) will depend upon whether constant returns to scale (CRS) or non-increasing returns to scale (NIRS) or variable returns to scale (VRS) are assumed. In this study, it is reasonable to anticipate that hospital size is more likely to be influenced by institutional or geographical constraints more than market environment, implying that a CRS assumption is likely to be tenuous. Thus, the less restrictive VRS assumption is specified. The VRS assumption also ensures that each hospital is compared with hospitals of similar size [11].

In the input-based model, efficiency measurement determines the minimum input (x*) that can produce an observed output bundle y^*t*^. Supposing that θ* represents the maximum contraction factor by which an input set x is adjusted in order to attain the minimum input level (x*), then x* = θ* *x*. Providing that (x*, *y*) lies within the feasible set above, the input-oriented measure of technical efficiency is given by,

TE(x,y)=x∗x=θ∗.
 MathType@MTEF@5@5@+=feaafiart1ev1aaatCvAUfKttLearuWrP9MDH5MBPbIqV92AaeXatLxBI9gBaebbnrfifHhDYfgasaacH8akY=wiFfYdH8Gipec8Eeeu0xXdbba9frFj0=OqFfea0dXdd9vqai=hGuQ8kuc9pgc9s8qqaq=dirpe0xb9q8qiLsFr0=vr0=vr0dc8meaabaqaciaacaGaaeqabaqabeGadaaakeaacqqGubavcqqGfbqrcqGGOaakcqqG4baEcqGGSaalcqqG5bqEcqGGPaqkcqGH9aqpdaWcaaqaaiabbIha4naaCaaaleqabaGaey4fIOcaaaGcbaGaeeiEaGhaaiabg2da9GGaciab=H7aXnaaCaaaleqabaGaey4fIOcaaOGaeiOla4caaa@3E65@

Thus, TE is formulated as *θ *= min *θ *: (*θ x*, *y*) ∈ *L*^*t*^.

In the health economics literature, the intuition behind formulating an input-based model is to model hospitals as input-minimisers rather than seekers of greater patient numbers. Hospitals in Zambia do not have much control over outputs. In this context, efficiency seeks to test whether a given hospital could have provided its largely exogenously-determined service profile with fewer resources.

Computationally, our short-run input-based technical efficiency is estimated by solving the following linear programming problem independently for each hospital [10].

*Minθ*

subject to constraints:

∑j=1Jzjymj≥yj
 MathType@MTEF@5@5@+=feaafiart1ev1aaatCvAUfKttLearuWrP9MDH5MBPbIqV92AaeXatLxBI9gBaebbnrfifHhDYfgasaacH8akY=wiFfYdH8Gipec8Eeeu0xXdbba9frFj0=OqFfea0dXdd9vqai=hGuQ8kuc9pgc9s8qqaq=dirpe0xb9q8qiLsFr0=vr0=vr0dc8meaabaqaciaacaGaaeqabaqabeGadaaakeaadaaeWbqaaiabdQha6naaBaaaleaacqWGQbGAaeqaaOGaemyEaK3aaSbaaSqaaiabd2gaTjabdQgaQbqabaGccqGHLjYScqWG5bqEdaWgaaWcbaGaemOAaOgabeaaaeaacqWGQbGAcqGH9aqpcqaIXaqmaeaacqWGkbGsa0GaeyyeIuoaaaa@3F9E@

∑j=1Jzjxmj≤θxnj
 MathType@MTEF@5@5@+=feaafiart1ev1aaatCvAUfKttLearuWrP9MDH5MBPbIqV92AaeXatLxBI9gBaebbnrfifHhDYfgasaacH8akY=wiFfYdH8Gipec8Eeeu0xXdbba9frFj0=OqFfea0dXdd9vqai=hGuQ8kuc9pgc9s8qqaq=dirpe0xb9q8qiLsFr0=vr0=vr0dc8meaabaqaciaacaGaaeqabaqabeGadaaakeaadaaeWbqaaiabdQha6naaBaaaleaacqWGQbGAaeqaaOGaemiEaG3aaSbaaSqaaiabd2gaTjabdQgaQbqabaGccqGHKjYOiiGacqWF4oqCcqWG4baEdaWgaaWcbaGaemOBa4MaemOAaOgabeaaaeaacqWGQbGAcqGH9aqpcqaIXaqmaeaacqWGkbGsa0GaeyyeIuoaaaa@42AB@

For all *n *= 1,..., *N*, *m *= 1,..., M, and *θ*, *z *≥ 0

With VRS, we have an additional (convexity) constraint [11];

∑j=1Jz=1.
 MathType@MTEF@5@5@+=feaafiart1ev1aaatCvAUfKttLearuWrP9MDH5MBPbIqV92AaeXatLxBI9gBaebbnrfifHhDYfgasaacH8akY=wiFfYdH8Gipec8Eeeu0xXdbba9frFj0=OqFfea0dXdd9vqai=hGuQ8kuc9pgc9s8qqaq=dirpe0xb9q8qiLsFr0=vr0=vr0dc8meaabaqaciaacaGaaeqabaqabeGadaaakeaadaaeWbqaaiabdQha6jabg2da9iabigdaXaWcbaGaemOAaOMaeyypa0JaeGymaedabaGaemOsaOeaniabggHiLdGccqGGUaGlaaa@37BF@

where:

*θ *= measures efficiency, or the factor by which inputs should be minimised in order to make each hospital produce at the efficient frontier,

z_j _= The z_j _-variables are called intensity variables and are used in the DEA model to identify efficient production,

x_nj _= amount of input *n *used by hospital *j*,

y_mj _= amount of output *m *produced by hospital *j*,

M = number of outputs,

N = number of inputs.

### Decomposition of Technical Efficiency

In this paper, we propose to decompose technical efficiency into scale efficiency and efficiency due to congestion [10]. Such a conceptual distinction is important because it helps to identify the areas for action. For example, addressing some sources of technical inefficiency may lie with planners at higher levels (e.g. level of hospital staffing or capitalisation), while other sources may be the responsibility of implementers at service delivery level.

#### Scale Efficiency

Scale efficiency has to do with a production unit operating at its optimal operating size given its output. An intuitive interpretation of scale efficiency is that, given its output level or external demand, there is a hypothetical scale of operations that makes each hospital most productive or efficient. The general theory is that when a firm becomes too big or too small, scale changes can lower costs and efficiency. Scale efficiency is health care industry is a consequence of market and institutional constraints which ensures that production units do not operate at optimal size. Scale efficiency is calculated by dividing a hospital's technical efficiency score under the assumption of CRS by its technical efficiency score under VRS.

Thus, the input-oriented measure of scale efficiency for the *j*^*th *^hospital is calculated by the following ratio[10]:

SEj=TEj(yj,xj;CRS,S)TEj(yj,xj;VRS,S).
 MathType@MTEF@5@5@+=feaafiart1ev1aaatCvAUfKttLearuWrP9MDH5MBPbIqV92AaeXatLxBI9gBaebbnrfifHhDYfgasaacH8akY=wiFfYdH8Gipec8Eeeu0xXdbba9frFj0=OqFfea0dXdd9vqai=hGuQ8kuc9pgc9s8qqaq=dirpe0xb9q8qiLsFr0=vr0=vr0dc8meaabaqaciaacaGaaeqabaqabeGadaaakeaacqWGtbWucqWGfbqrdaWgaaWcbaGaemOAaOgabeaakiabg2da9maalaaabaGaemivaqLaemyrau0aaSbaaSqaaiabdQgaQbqabaGccqGGOaakcqWG5bqEdaWgaaWcbaGaemOAaOgabeaakiabcYcaSiabdIha4naaBaaaleaacqWGQbGAaeqaaOGaei4oaSJaem4qamKaemOuaiLaem4uamLaeiilaWIaem4uamLaeiykaKcabaGaemivaqLaemyrau0aaSbaaSqaaiabdQgaQbqabaGccqGGOaakcqWG5bqEdaWgaaWcbaGaemOAaOgabeaakiabcYcaSiabdIha4naaBaaaleaacqWGQbGAaeqaaOGaei4oaSJaemOvayLaemOuaiLaem4uamLaeiilaWIaem4uamLaeiykaKcaaiabc6caUaaa@5897@

A hospital's score will be one if it is scale-efficient or less than one otherwise. Further, for scale-inefficient hospitals, characteristics of inefficiency are identified by economies of scale (increasing returns to scale) if *TE*_*j *_(*VRS*) { *TE*_*j *_(*NIRS*), while *TE*_*j *_(*VRS*) { *TE*_*j *_(*NIRS*) signal diseconomies of scale. The Onfront^® ^programme, which has been used for estimating efficiency in this paper, permits this calculation.

#### Inefficiency due to Congestion

Fare et al [10] first proposed a method for analysing input congestion in efficiency measurement using the disposability property. Input congestion arises if an input cannot be profitably utilised in the production process. For instance, excess labour or capital which cannot be disposed of will reduce overall productivity. The input-based measure of congestion efficiency (CE) is defined as a ratio of the technical efficiency (TE) measure under strong disposability of inputs (TE(VRS,S)) to the TE measure under weak input disposability technology (TE(VRS,W)). Thus [10]:

CEj=TEj(yj,xj;VRS,S)TEj(yj,xj;VRS,W).
 MathType@MTEF@5@5@+=feaafiart1ev1aaatCvAUfKttLearuWrP9MDH5MBPbIqV92AaeXatLxBI9gBaebbnrfifHhDYfgasaacH8akY=wiFfYdH8Gipec8Eeeu0xXdbba9frFj0=OqFfea0dXdd9vqai=hGuQ8kuc9pgc9s8qqaq=dirpe0xb9q8qiLsFr0=vr0=vr0dc8meaabaqaciaacaGaaeqabaqabeGadaaakeaacqWGdbWqcqWGfbqrdaWgaaWcbaGaemOAaOgabeaakiabg2da9maalaaabaGaemivaqLaemyrau0aaSbaaSqaaiabdQgaQbqabaGccqGGOaakcqWG5bqEdaWgaaWcbaGaemOAaOgabeaakiabcYcaSiabdIha4naaBaaaleaacqWGQbGAaeqaaOGaei4oaSJaemOvayLaemOuaiLaem4uamLaeiilaWIaem4uamLaeiykaKcabaGaemivaqLaemyrau0aaSbaaSqaaiabdQgaQbqabaGccqGGOaakcqWG5bqEdaWgaaWcbaGaemOAaOgabeaakiabcYcaSiabdIha4naaBaaaleaacqWGQbGAaeqaaOGaei4oaSJaemOvayLaemOuaiLaem4uamLaeiilaWIaem4vaCLaeiykaKcaaiabc6caUaaa@58A5@

## Data

### Sampling and hospital characteristics

Data collection involved field visits to individual hospitals as hospitals do not consistently report data to MOH headquarters. An atlas of hospital facilities in Zambia showed that in 2003 (the time of the survey) there were 97 hospitals with a total of 28,940 beds and cots between them. Five of these are third level (or tertiary) hospitals, 18 are second-level while 74 are classified as first-level hospitals. In terms of ownership, church missions own six of the second-level hospitals and 21 of first-level hospitals, while the private sector owns 17 of the first-level hospitals. The rest are owned by the Government [12].

Although these hospitals are classified along a hierarchical three-tier referral system, in reality the level of complexity of services between these hospitals is less significant [6]. In terms of the resources and infrastructure in these hospitals, the distinction between a first-level hospital and a second-level hospital is in many cases even less clear. Basically, these hospitals provide what is referred to as first- and second-level referral services. That is, they receive patients who have been referred from a network of health centres (as well as patients that have bypassed lower level facilities).

Operationally, these hospitals house four main units in internal medicine, surgery, paediatric care and gynaecology and obstetric care, in which they offer major diagnostic and therapeutic services. Typically, interventions conducted would include surgical procedures including caesarean sections, eye and ear operations, repair of fractures and injuries, and so on. Other examples of care provided include management of diabetes, hypertension, severe malaria, many HIV/AIDS opportunistic infections, palliative care for severely undernourished and sick children, and so on. More specialised care such as cardiac care is referred to the only teaching hospital in the country. By and large, these hospitals provide a comparable range and quality of services.

Further, referrals do not often happen according to the pattern that would be predicted by this formal classification. For example, Livingstone General hospital does refer to Monze mission although in theory this is not supposed to happen. Chipata General hospital also refers patients to Saint Francis which is of the same level, as does Kitwe Central to Ndola Central. However, what we also learnt is that in large part these hospitals are offering first-level care. Most second-level hospitals lack the skilled staff and equipment to perform most second-level services [6,7].

It appears that the location of hospitals in Zambia has largely been a result of strategic decisions by the Government in cooperation with the decisions of the former mining conglomerate and the various Church Missions. The government tended to build hospitals in areas where mission or mine hospitals did not exist and vice-versa. As a consequence of this 'location-allocation' exercise, each district would typically have either a Government or a Mission hospital and not both. Further, most districts typically have either a second-level or a first-level hospital, not both. As a result, a good proportion of their activity profile is dedicated to first-level care. This partly explains why hospitals are quite similar functionally, despite their tier classifications. A comprehensive study on hospitals and the referral system in Zambia can be found elsewhere [6].

A sample size of 32 hospitals was determined based on the budget. This would cover about 30% of first- and second-level hospitals. Further, it was considered that the survey be conducted in five provinces (Central, Copperbelt, Eastern, Southern and Central) of the nine provinces of Zambia in order to ensure there was contiguity between the study sites. Two hospitals were removed for incomplete data, leaving a sample of 30. This sample was composed of 18 (60%) Government-owned hospitals, eight (27%) Church mission hospitals and four(13%) private hospitals. In terms of geographical location, 16 (53%) hospitals are based in rural areas.

### Specification of inputs and outputs

An important step in efficiency analysis is to specify outputs. One of the contentious issues in efficiency in health care is the use of intermediate or so called 'process' outputs. It is argued that the output of interest against which hospital activity should be assessed is patients' health gain. The basic concern is that there should be a way to discount hospital service outputs to the extent that they are of such poor quality that they do not confer any health gains on patients. Though desirable, such an idea would be very difficult to implement, if not impracticable, in many developing countries. As a compromise, an intermediate level output, namely hospital service, which supposedly improves patients' health, is used, sometimes with some adjustment for quality or case-mix differentials across hospitals [13].

However, quality adjustment in outputs could not be performed in this study due to non-availability of the necessary data on case-mix and quality. This means that we have assumed that the case mix and severity patterns were constant across the sample hospitals. This has been applied elsewhere in the literature [14-17]. In a study of American hospitals, Grosskopf and Valdmanis [18] found no significant differences in efficiency scores in a comparison between two models that applied case-mix adjusted and unweighted output variables. Neither did we have patient-level data to apply some type of quality-adjustment based on differential mortality rates across hospitals as a measure of quality. Nonetheless, our output set is comparable to those specified in many other studies [19]. The four output measures used are presented in Table 1.

**Table 1:** Input and output definitions

*Variable*	*Definition*	*Unit of measurement*
Inputs		
X1	Non-labour cost	Non-labour expenditure
X2	Medical Doctors	Number of staff
X3	Nurses +Cos+Labtechs+ Radiographers+pharmacists	Number of staff
X4	Administrative and other staff	Number of staff
Outputs		
Y1	Ambulatory care	No. of visits
Y2	Inpatient	No. of Bed-days
Y3	MCH	No. of deliveries
Y4	Lab tests+X rays+Theatre operations	No. of tests or operations performed

On the input side, we have labour and non-labour inputs as resources that go into the production of hospital services. Labour inputs were constructed in terms of numbers of personnel. This specification of the labour input can be problematic as many health staff in developing countries can spend time away from duty without being accounted for. However, attempts to calculate the time that staff spend on duty (total hours worked) proved unsuccessful as most human resource departments did not keep a usable duty roster.

Thus, four input variables included were, total non-labour cost (*x*_1_), number of medical doctors (*x*_2_), number of nursing and other clinical staff (*x*_3_) and number of non-clinical staff (*x*_4_). It was confirmed during the field visits that there is a good degree of substitutability among clinical staff especially in rural hospitals because of staff shortages. The input x1 was a composite including running costs, administration, allowances, overhead costs and capital costs. To estimate x1, all capital and equipment costs were annualised using a life span of 30 years for buildings and 10 years for vehicles and equipment, and a discount rate of 5 %. These rates have been applied in studies in Africa [20].

### Descriptive Statistics of input and output data

Summary statistics of the variables of interest are presented in Table 2. This is intended to provide a general description of the resource endowment and output set of hospital sample. The high standard deviations reflect that the data were gathered from a sample of different size hospital units. The data shows that Zambian hospitals do have a considerable share of outpatient care in their activity portfolio. Further, the unfavourable patient-to-staff ratios that exist in Zambian hospitals can be discerned from the summary data.

**Table 2:** Descriptive statistics of model variables

Variable	Arithmetic Mean	Std. Dev.
Y1: Visits	24687.2	14246.2
y2: Inpatient days	39888.6	36229.6
y3: Deliveries	1176.5	1437.2
y4: Investigative procedures	49329.4	68174.5
x1: # of Doctors	14.8	14.1
x2: Nurses+Cos+Labtechs+Radiographers+pharmacists	93	84.1
x3: Administrative and other staff	12.8	14.9
X4: Non-labour expenditure, Kwacha (US$)	K2071994690 (460,443.26)	K3445010760 (765,557.95)

## Results and Discussion

### Efficiency estimates

Estimated efficiency scores are presented in Table 3. The results show that eleven of the thirty hospitals (40% of sample) are efficient. These hospitals form the best practice frontier. This result means that majority of the hospitals are inefficient, i.e., are not operating at the technically efficient levels. According to these results, the average relative efficiency level is modest at 67 %. Further, the median score indicates that 50% of hospitals had an efficiency score of 66% or less. This result indicates that, collectively, hospitals could produce their current output levels with a 33% reduction in the resource inputs included in this model. We also note that the average score for the 18 inefficient hospitals is 42%, implying that these hospitals could double their outputs to reach the frontier formed by their efficient counterparts in the sample. Overall, this result suggests that substantial resources could be saved if all hospitals were to operate efficiently. Studies in other African countries have reported technical efficiency of this order [21-25].

**Table 4:** Input-based measure of technical efficiency for each hospital (with number of times a hospital has been used as a reference unit)

Hospital	*Ownership*	*Fi *(*y,x | V,S*)
1. Konkola Hospital	Private	1.00 (3)
2. Nyimba Hospital	Government	1.00 (8)
3. Wusakile Hospital	Private	1.00 (5)
4. Kabwe Mine	Government	1.00 (2)
5. Ndola Central	Government	1.00 (2)
6. Livingstone General	Government	1.00 (1)
7. St Francis Mission	Mission	1.00 (7)
8. Lumezi Mission	Mission	1.00 (19)
9. Kabwe General	Government	1.00 (2)
10. Kitwe Central	Government	1.00 (2)
11. Mazabuka District	Government	1.00 (2)
12. Minga Mission	Mission	1.00 (2)
13. Monze Mission	Mission	0.68 (0)
14. Kalomo District	Government	0.67 (0)
15. Sinozam Friendship	Private	0.67 (0)
16. Nchanga South	Private	0.65 (0)
17. Nchanga North	Government	0.60 (0)
18. St Theresa Mission Hospital	Mission	0.58 (0)
19. Zimba Mission	Mission	0.53 (0)
20. Gwembe District Hospital	Government	0.41 (0)
21. Chipata General	Government	0.39 (0)
22. Kalulushi District Hospital	Government	0.39 (0)
23. Petauke District Hospital	Government	0.38 (0)
24. Mwami Mission	Mission	0.37 (0)
25. Lundazi District Hospital	Government	0.33 (0)
26. Thompson Hospital	Government	0.32 (0)
27. Chikankata Mission	Mission	0.30 (0)
28. Arthur Davison	Government	0.29 (0)
29. Kamuchanga Hospital	Government	0.27 (0)
30. Choma General	Government	0.23 (0)

Efficiency scores for individual hospitals are presented in Table 4. Compared with Kitwe Central hospital, Thompson hospital for example could produce the same output level (or activities) with only 30% of its current resources. It can also be noticed that there are considerable variations in efficiency scores between hospitals. For example, the most efficient hospitals are over four times as efficient as the least efficient. The most inefficient hospital (Choma) has an efficiency score of only 23%. In a DEA framework, high variability in observed performance across a sample provides strong prima facie evidence that the Zambian health system suffers significant losses in resources (and potentially health benefits to patients) due to operating inefficiency. (Table 3)

Also indicated in the brackets in Table 4 is the number of times each efficient hospital has been used as a reference hospital for itself as well as for others with a similar input-output mix. Onfront^® ^identifies the hospitals which have been referenced with each hospital on the frontier thereby facilitating comparison. For example, Mazabuka is shown to be a reference hospital for Petauke hospital. This implies that Petauke hospital only reaches about 40% of the efficiency of its peer, Mazabuka hospital. Lumezi hospital has been used as a reference for 19 hospitals. The estimation procedure has recognised that these 19 hospitals do have some service profile that is similar to Lumezi hospital. This type of information facilitates further investigation of hospital characteristics, operating practices and other attributes.

### Scale efficiency analysis

Scale efficiency tests indicate that a hospital may be operating at activity levels that are contributing to higher than minimum-average costs (or most productive scale size). This seems to suggest that while on one hand some hospitals may be operating at too large a scale to maximise the productivity of their inputs, other hospitals appear to be too small and therefore exhibiting higher average costs. Summary and individual hospital scale efficiency scores are presented in Table 5.

**Table 5:** Scale efficiency summary statistics

*Parameter*	*Score*
Mean score	.80
Median score	.89
Standard Deviation	.33
Least Scale efficiency Score	.19
% of scale efficient Hospitals	13.3
% of hospitals exhibiting increasing returns to scale	43.3
% of hospitals exhibiting decreasing returns to scale	43.3

The average scale efficiency score of all the hospitals is 80%. Our results further show that only 4 (13%) out of 30 hospitals were operating at optimal plant size, though many others are operating very close to their optimal size (Table 6). Further, by examining individual hospital efficiency scores, we are able to furnish the nature of scale inefficiency, i.e. determine whether an individual hospital is operating in an area of increasing or decreasing returns to scale.

Interestingly, the pattern of scale inefficiency indicates that about 43% of hospitals were operating on increasing to scale while another 43% were operating in a range of decreasing returns to scale. In Zambian context, scale inefficiency is a matter of planning rather than management failures [6]. This suggests that downscaling hospitals exhibiting decreasing returns to scale and shifting resources towards those facing increasing returns to scale would generally yield efficiency gains. Four big hospitals namely, Chipata General, Kabwe General, Livingstone General and Ndola Central all exhibit decreasing returns to scale. In the case of Ndola Central, which had a VRS efficiency score of 1, this result implies that even if they perform efficiently with their inputs, maintaining their current capacity casts the hospital into a region of considerable technical inefficiency, from a CRS benchmark (*cf*. equation 2).

Economics suggests that scale efficiency, which results from finding the minimum average cost level of operation, is not really a short run phenomenon. In other words, changing the staffing profile and capital stock of a hospital cannot be done within a short period of time. Improving scale efficiency will require 'right-sizing' hospitals in line with their output profile. This would require careful planning.

### Inefficiency attributable to input congestion

Estimates of congestion following equation (3) show the following results. The results in Table 7 show that congestion is contributing to inefficiency in 18 of the hospitals. It can also be noted that some of the hospitals that were found to be operating at optimal scale were technically inefficient because of using too much of some input or inputs. The average congestion efficiency score was 81%. A hospital such as Nchanga North with a congestion efficiency score of 60% can contract its excessive inputs by 40% without worsening outputs. For instance, if there are too many workers relative to other inputs, costs can be saved by transferring some staff to other units, or declaring their posts redundant.

**Table 7:** Congestion scores for each hospital

Hospital	Congestion measure
1. Konkola	1.00
2. Wusakile	1.00
3. Mazabuka	1.00
4. Livingstone	1.00
5. Kitwe Central	1.00
6. Ndola Central	1.00
7. Kabwe Mine	1.00
8. Kabwe Gen	1.00
9. Lumezi	1.00
10. St Francis	1.00
11. Minga	1.00
12. Nyimba	1.00
13. Zimba	0.96
14. Kamuchanga	0.93
15. Petauke	0.90
16. Chikankata Mission	0.88
17. Gwembe	0.85
18. Arthur Davison	0.83
19. Choma	0.74
20. Kalulushi	0.74
21. Ibenga Mission	0.69
22. Monze Mission	0.68
23. Kalomo	0.67
24. Sinozam Friendship(NM)	0.67
25. Nchanga South	0.65
26. Nchanga North	0.60
27. Mwami	0.59
28. Chipata Gen	0.39
29. Lundazi	0.33
30. Thompson	0.32

### Some statistical issues

A limitation of the standard DEA estimator is that it does not render itself readily to statistical inference procedures about the true efficiency (e.g. confidence intervals and hypothesis testing). A common approach in the literature has been to model the DEA efficiency score as a function of a set of covariates in a second-stage regression. However, this approach is likely to produce misleading inferences for at least two reasons. First, Simar and Wilson [26] have observed that since the DEA estimator gives relative efficiency scores, the efficiency estimates of observations on the frontier are highly likely to be correlated with those of other observations. They argue that in many empirical situations, this would lead to a complex-order serial-correlation problem, thereby rendering inference from standard regressions invalid. Further, a less severe problem is that by the distribution of DEA scores, it is likely that the normality assumption would be violated.

A non-parametric Mann-Whitney test was used to test whether location and ownership have an effect on performance (no data was available by hospital catchment on other traditional environmental factors such as socio-economic status, epidemiology, etc.). The null hypothesis is that the DEA score of government-owned hospitals is equal to the DEA score of private and mission hospitals. In the case of location, the null was that the DEA score for urban-based hospitals is equal to the score for rural hospitals. In both cases, probability values *P *= .1025 and *P *= .2702 with regard to ownership and location respectively, point to a failure to reject the null hypotheses that both ownership and location did not have a significant effect on efficiency.

Confidence intervals are often generated using the bootstrapping (BS) method, based on non-parametric simulation of the underlying data. The BS method tests the sensitivity of efficiency to sampling variation of the frontier. This is implemented by re-sampling numerous times from the empirical data. In the literature algorithms for performing valid bootstrapping are still under development [27,26]. In this study, a basic bootstrap algorithm which samples from estimated DEA scores was implemented to generate confidence intervals around the original DEA mean estimate, without re-estimating efficiency scores [28]. A drawback of this procedure is that it produces narrower confidence intervals, which increases the chance of type one error [28].

With 1000 replications, the BS mean DEA efficiency score is 0.657, implying that the original sample mean DEA efficiency score reported in table 3 was biased upwards by a magnitude of .014 (bias is defined as the BS-score minus original score). Our BS 95% confidence interval is; (0.5525, 0.7625). These tests were done in STATA 9.2.

**Table 3:** DEA results summary

Statistic	Value
Mean efficiency score	0.67
Median efficiency score	0.66
Standard Deviation	0.33
Least Efficiency Score	0.23
Mean efficiency score Government owned	0.63
Mean efficiency score mission and private owned	0.73

### Study limitations and suggestions for future work

This study suffers from some important limitations upon which future studies should improve. One of the main limitations is the small sample size which limits the generalisability of study findings. A large sample size is desirable because DEA results can be sensitive to sample size. Future studies should explore analysis with bigger sample sizes. Further, we are unable to determine how much of the inefficiency might be caused by any systematic quality variations. Due to the poor quality of data-keeping systems in Zambia the study was unable to obtain data that would have been helpful in defining quality-adjusted outputs. A bigger sample size would also permit a more rigorous characterisation of hospital outputs. More specifically, greater effort should be devoted to collecting more detailed output data that might improve the quality of future efficiency studies. Finally, more work is needed in developing methods for deriving appropriate performance quality indicators.

## Conclusion

In the current economic situation of Zambia's health sector, it might be unfashionable to emphasise efficiency as a policy objective. This paper takes the view that the prospects for Zambia to attain its health goals depend in a significant way on how efficiently resources are utilised. Using a simple empirical model, we demonstrate the current levels and patterns of operating inefficiency of public health facilities. According to our results, Zambian hospitals could have attained their output levels with about 30% less resources, suggesting potential for better service coverage. For instance, if inefficiency is eliminated or minimised, the extra resources could be invested in a range of operational areas such as better quality patient care, new technology, expansion of service profile, staff training in needed specialties or improved staff welfare. Hospitals could also finance part of their debt-stock.

Further, decomposing technical efficiency distinguished the role of scale and input congestion in contributing to hospital inefficiency. In particular, unsuitable hospital scale of operation or size and low productivity of some inputs reinforce each other to make Zambian hospitals technically inefficient at delivering services. In this case, strategies such as hospital mergers or down-grades may help bring down costs and improve overall efficiency in the hospital industry. It is possible that some hospitals may be using more of some resources only because they have been historically over-funded or over-staffed relative to their outputs.

However, it is important to emphasise that with the paucity of good quality data on inputs and outputs, these results are more indicative than definitive measures of efficiency. They point to 'trouble spots' which could be a starting point for further investigation. Data required for this purpose should be included in routine data-collection platforms such as health management information systems and national health accounts.

Finally, in order to make the most of the increasing global resources flowing into the health sector, all forms of current systemic weaknesses and waste have to be eliminated or greatly minimised. Technical inefficiency is an example of such weaknesses. For this to happen, policy makers would need to assign significant priority to rigorous forms of system performance assessment. In the future, when some of the current generous global financing initiatives lie in the past, the Ministry of Health and the Zambian public would not wish to rue this as yet another missed opportunity.

## Competing interests

The author(s) declare that they have no competing interests.

**Table 6:** Scale efficiency score and returns-to-scale characteristics of each hospital

Hospital	No. of beds and cots	Scale Efficiency score	Type of scale inefficiency
Kalomo	52	0.49	IRS
Kamuchanga	60	0.78	DRS
Gwembe	68	0.19	IRS
Nyimba	73	0.65	IRS
St. Theresa's Mission	93	0.97	DRS
Sinozam	93	0.89	IRS
Kabwe Mine	101	0.77	IRS
Thomson District	115	0.96	IRS
Lumezi	117	1.00	No scale inefficiency
Konkola Mine Hospital	125	1.00	No scale inefficiency
Kalulushi Mine	132	0.66	IRS
Minga Mission	133	0.95	IRS
Lundazi District	138	0.45	IRS
Zimba Mission	141	0.60	IRS
Mazabuka	142	1.00	No scale inefficiency
Petauke	142	0.98	DRS
Nchanga South	171	0.96	DRS
Mwami Mission	187	0.89	IRS
Choma General	218	0.99	IRS
Arthur Davison	250	0.78	DRS
Nchanga North	293	0.96	DRS
Monze Mission Hosp.	322	0.98	IRS
St. Fra ncis	455	0.95	DRS
Wusakile Hospital	478	1.00	No scale inefficiency
Livingstone	501	0.58	DRS
Kabwe	515	0.36	DRS
Chipata General	536	0.75	DRS
Chikankata	540	0.98	DRS
Kitwe Central	645	0.88	DRS
Ndola Central	948	0.56	DRS

## Pre-publication history

The pre-publication history for this paper can be accessed here:


